# Cost-effectiveness of GnRH antagonist implementation on hCG injection day

**DOI:** 10.4274/tjod.galenos.2019.56255

**Published:** 2019-03-27

**Authors:** Ayşe Zehra Özdemir, Bülent Ayas, Davut Güven, Aysın Pınar Türkmen, Çağrı Gülümser

**Affiliations:** 1 Ondokuz Mayıs University Hospital, In vitro Fertilisation Center, Samsun, Turkey; 2 University of Health Science, Department of Obstetrics and Gynecology, İstanbul, Turkey

**Keywords:** Controlled ovarian stimulation, cost effectiveness, pregnancy rates

## Abstract

**Objective:**

To compare the outcomes of antagonist stimulation protocols and to compare the cost effectiveness.

**Materials and Methods:**

Between 2011 and December 2017, a total of 354 women who underwent intracytoplasmic sperm injection and controlled ovarian stimulation with antagonist protocols were enrolled in the study. The antagonist implementation on the day of human chorionic gonadotropin (hCG) was continued for 194 of women, whereas the antagonist was stopped 36 hours before in 160 women. The stimulation outcomes of patients and cost-effectiveness of the regimens were compared.

**Results:**

There was a significant difference between the groups in terms of number of cryopreserved embryos, mature/immature oocyte ratio, and embryo transfer cancellations (p<0.05). The median value for the mature/immature oocyte ratio was 1.1 (0.2-7.5) and 1 (0.5-15) (p=0.001), and the ET cancellation was 5.3% vs. 1% for group 1 and 2, respectively (p=0.037). There was no difference between the groups in terms of pregnancy rates (p=0.197).

**Conclusion:**

No difference was found in the clinical pregnancy rates between the two groups. For this reason, the cessation of antagonist implementation on the day of hCG seems more advantageous in terms of cost-effectiveness and fewer injections.

**PRECIS:** Cessation of antagonist implementation on the day of hCG seems more advantageous in terms of cost-effectiveness without an effect on clinical pregnancy rate.

## Introduction

Gonadotrophin-releasing hormone (GnRH) antagonists have been used since 1999 in to prevent the luteinizing hormone (LH) peak in controlled ovarian stimulation^(1,2)^. GnRH antagonists suppress the release of follicle-stimulating hormone (FSH), and especially that of LH by competitively blocking the GnRH receptors in the anterior pituitary. Its effect starts rapidly and then rapidly reverts when the medication is stopped^(3)^. When compared with GnRH agonists, it is widely used as a safer, time-efficient, and more affordable stimulation model. In the first meta-analyses conducted on GnRH antagonists, the estrogen level and total number of oocytes on the human chorionic gonadotropin (hCG) day were found to be lower than with the agonists^(4)^. The pregnancy rates were determined to be slightly lower^(5)^. For this reason, many modifications have been made in the standard antagonist protocol in order to improve the efficiency of stimulation incorporating GnRH antagonists.

In recent studies^(6)^, no difference was found between GnRH antagonists and GnRH agonists in terms of live birth rates. These recent advances suggest that the success of therapy increases as the experience with the use of antagonist increases. However, there is still no standard antagonist protocol, and significant effort is made in order to minimize the negative effects of antagonists by decreasing the number of antagonist injections.

The negative effects of GnRH antagonists are thought to originate from decreasing the LH level below the critical threshold that ensures the development of follicles. However, LH increases the aromatase activity in follicular development by having a synergistic interaction with FSH, and thus the estrogen secretion increases and ovulation and luteinization are ensured^(7)^. LH increase is necessary for final oocyte maturation. There are studies reporting that antagonist implementation on the hCG day might have a negative effect on the maturation of the final oocyte^(7,8)^. It was thought that antagonists had characteristics that negatively affected follicular development because the effects revert rapidly if the antagonist is not applied on the day of hCG.

We aimed to compare the stimulation outcomes of patients who did and did not receive antagonist on the day of hCG, and to contribute to the optimal stimulation protocol especially aspects of costs saving and reduce injection.

## Materials and Methods

This is a retrospective study. Ethics committee approval was obtained from Ondokuz Mayıs University (2018/164). All subjects gave their written informed consent. Between January 2011 and December 2017, a total 354 women underwent intracytoplasmic sperm injection (ICSI) and controlled ovarian stimulation with an antagonist protocol at Ondokuz Mayıs University Reproduction Unit. Patients aged between 18 and 40 years with regular menstruation and no endocrinologic disease were enrolled in the study. The exclusion criteria were as follows: severe male factor such as oligoasthenoteratozoospermia, patients with hydrosalpinx, endometriosis, and polycystic ovary syndrome, frozen cycles, and those with no oocyte in oocyte pick up (OPU) and no follicle development in induction (no LH or progesterone increase was observed among these patients). The antagonist implementation on the day of hCG was continued for 194 of patients (group 1), whereas the antagonist was stopped for 160 participants 36 hours before hCG injections (group 2). The rationale behind this approach was to reduce the number of injections and cost. We routinely administer the GnRH antagonist in the morning so that when we administer the GnRH antagonist on the hCG day it is administered 12 hours before hCG. When we skip the GnRH antagonist we administer GnRH antagonist 36 hours before hCG.

Embryo transfer (ET) cancellation was defined as patients with no developing embryos or living embryos on the day of ET. The patients were examined on the second or third day of menstruation, and the gonadotropin FSH (Gonal-F, Serono) implementation was applied at personal doses by considering the patient’s age, body mass index, and antral follicle count. GnRH Antagonist (0.25 mg cetrorelix acetate, Cetrotide, Serono) was added when the diameter of follicle reached 12 mm. When the diameters of two or more follicles reached 17 mm, recombinant hCG (Ovitrelle 250 mcg, Serono) was administered. OPU was performed 36 hours later following hCG administration and then the ICSI was performed. The ET was performed on the 3^rd^ day of OPU. The oocyte maturation was classified as metaphase 2, intermediate, and germinal vesicles in terms of cumulus/corona morphology, cytoplasmic clarity, zona thickness, and extent of perivitelline space values. The embryonic morphology was classified into 4 grades in terms of the regularity of blastomeres, the percentage of anucleate fragments, and all dysmorphic characteristic of the embryos. Grade I: 0% anucleate fragments, regularity of blastomeres, and no apparent morphologic abnormalities; grade II: <10% anucleate fragments, regularity of blastomeres, and no apparent morphologic abnormalities; grade III: 10% to 50% anucleate fragments, irregularity of blastomeres, and no apparent morphologic abnormalities; and grade IV: ≥50% anucleate fragmentation, irregularity of blastomeres, and apparent morphologic abnormalities.

Clinical pregnancy was defined as the detection of an intrauterine gestational sac. Each patient was given 100 mg progesterone intramuscular (Progestan 50 mg, Kocak) and 6 mg estradiol orally (Estrofem 2 mg, Novo Nordisk) as luteal support from the day of OPU. The serum specimen was taken from the patients on the morning of the hCG administration day, and the LH, progesterone, and estradiol (E2) measurements were performed.

The premature LH rise was defined as 10 mIU/mL, whereas the premature progesterone rise was defined as 1 ng/mL. The combined rise of LH and progesterone was considered as premature luteinization.

### Statistical Analysis

Statistical analysis was performed using the SPSS V23 software package (IBM Corp. Released 2015. IBM SPSS Statistics for Windows, Version 23.0. Armonk, NY: IBM Corp.). The normality of data distribution was tested using the Kolmogorov-Smirnov test. Non-normally distributed data were analyzed using the Mann-Whitney U test. Categorical data were analyzed using the chi-square test. The results were expressed as median (min-max) and frequency (percentage). The level of significance was set as p<0.05.

## Results

The mean age was 30±4 years. The mean follicle count was 10 and 11 in groups 1 and 2, respectively. No statistically significant difference was observed between the groups in terms of age, number of follicles, and FSH values (Table 1, p>0.05). The most frequent reason for infertility was unexplained infertility in both groups (group 1, 54% and group 2, 63%). Poor ovarian reserve was 12.5% and 11.3%, and male factor was 33.1% and 24.7% in groups 1 and 2, respectively. There was no statistically significant difference between the groups regarding the reasons of infertility (Table 2, p=0.165).

There were no statistically significant differences between the groups in terms of estrogen *(*p=0.066), progesterone (p=0.127), LH (p=0.636), number of collected oocytes (p=0.088), 2PN (p=0.193), number of M2 oocytes (p=0.515), total dose of gonadotropin used in stimulation (p=0.336), and endometrium thickness (p=0.656). However, we found a significant relationship between the groups regarding the number of cryopreserved embryos, ratio of mature/immature oocytes, number of transferred embryos, and ET cancellation (p<0.05). The median mature/immature oocyte ratio was found as 1.1 (0.2-7.5) and 1 (0.5-15) in group 1 and 2, respectively (p=0.001). ET cancellation was decided in 1% and 5.3% of women in group 1 and group 2, respectively (p=0.037). Premature ovulation or premature luteinization was not seen in any woman (Table 3).

Among the patients receiving antagonist on the day of hCG, the oocyte morphology was deteriorated in 4 patients with cycle cancellation. Extensive granulation was observed in the cytoplasm and no embryo could be achieved. Embryo development was arrested in the other 5 patients. Embryo grades were statistically similar between the groups (p=0.924) (Table 4). Forty-five (45/160, 28.1%) women in group 1 and 43 (43/194, 22.2%) in group 2 became pregnant. No significant difference was observed between the groups in terms of the pregnancy rates (p=0.197) (Table 5). Ovarian hyperstimulation syndrome (OHSS) was diagnosed in 30 and 21 woman in groups 1 and 2, respectively (p=0.059).

All women in group 1 received one less GnRH antagonist injection. The current cost of one antagonist injection is 25,714 USD. Among the total 194 women in group 1, the cost saving was about 5003 USD compared with group 2.

## Discussion

The rate of early LH surge is 20% in cycles in which no GnRH analog is used^(9)^. Thus, fewer oocytes are collected, fewer embryos form, and pregnancy rates decrease. GnRH analogs are widely used for preventing the increase of premature LH. In the literature, it was reported that the pregnancy rates of patients receiving controlled ovarian stimulation using an antagonist protocol were lower when compared with agonist cycles^(6)^. However, when compared with GnRH agonists, GnRH antagonists had significant advantages such as shorter ovarian stimulation duration and formation of fewer OHHS^(10)^. For this reason, it is aimed to increase pregnancy rates by applying the minimum antagonist dose that can prevent the premature increase of LH in order to protect from potential negative effects of antagonist agents.

The reason for collecting fewer oocytes and determining lower E2 levels in cycles in which the GnRH antagonists were applied might be the suppression of GnRH antagonists by decreasing LH levels below the critical threshold^(4)^. In natural and induced cycles, there is a phenomenon called the LH window^(7)^. In folliculogenesis, a certain level of LH is necessary for the proper development of oocyte. Otherwise, when the level of LH exceeds a certain limit, the aromatase activity and cellular growth are inhibited^(7)^. Applying competitive blockage, GnRH antagonists suppress the LH level, in addition to FSH, below the critical threshold required for follicular development.

However, GnRH antagonists suppress the pulsatile secretion of LH for 456 minutes^(11) ^and we aimed to systematically collate evidence on the clinical efficacy of GnRH agonist triggering in patients undergoing assisted reproduction in GnRH antagonist protocols. Twenty-three publications were identified by a comprehensive literature search that included PubMed, Embase and the Cochrane Library. Three publications out of 23 fulfilled the inclusion criteria for meta-analysis, which were (i. For this reason, when no GnRH antagonist is applied on the day of hCG, this effect would disappear. This short-run LH increase will ensure more proper final oocyte maturation but not cause luteinization^(7)^ because the process of luteinization requires time.

The antagonist given at double-dose before the day of hCG in order to block the OHSS development decreases the estrogen levels but does not affect pregnancy outcomes^(12)^. This outcome might be because the dose increase was not applied in the critical process of oocyte maturation. The shorter half-life of GnRH antagonists might have contributed to this result.

Another mechanism through which the GnRH antagonists can block oocyte maturation is the direct effect on the ovary through the GnRH receptors existing on granulosa cells^(13,14,15)^. In animal studies, it was determined that GnRH analogs were effective on the ovarian functions of in vitro granulosa luteal cells such as steroidogenesis, oocyte maturation, and follicle rupture^(16)^.

In the present study, the mature/immature oocyte ratio of patients receiving no antagonist on the day of hCG was found to be statistically significantly higher when compared with patients receiving no antagonist on the day of hCG. Chang et al.^(8)^ reported that the rate of mature oocytes increased significantly among patients receiving no GnRH antagonist on the day of hCG. However, Chang et al.^(7)^ also performed a prospective study on 86 patients. They compared patients receiving GnRH antagonist on the day of hCG and those receiving no GnRH antagonist, and they reported that the controlled ovarian hyper-stimulation results were similar. When follicles reaching a size greater than critical threshold were detected, final oocyte maturation was achieved under the effect of hCG. We believe that the antagonist implementation on the day of hCG deteriorates the final oocyte maturation because of the results we achieved.

Different from other two studies^(7,8)^, we determined that ET cancellation was statistically significantly higher among patients receiving antagonist on the day of hCG. The oocyte morphology was found to be deteriorated among 4 of these patients. Extensive granulation was observed in the cytoplasm. In the resting 5 patients, embryonic developmental arrest was observed. However, in the group that stopped receiving antagonist, two patients who were incapable of forming an embryo were found.

Embryo arrest is due to maternal factors^(17)^. The transcription factors of the oocyte affect the cleavage of the embryo in the early stages. These factors are formed during oogenesis. Embryos that contain low maternal transcription factors in the early cleavage stage arrest in an inappropriate microenvironment^(18)^. In our study, the arrest of the embryo in the group 2 patients may be due to the negative effects of GnRH antagonist during oocyte maturation.

The oocyte quality indicates the fertilization potential. In the present study, the increase in the mature oocyte rate among patients receiving no antagonist on the day of hCG and the lower level of fertilization failure are in harmony with each other. Antagonist implementation on the day of hCG may cause lower quality and fewer mature oocytes by deteriorating the microenvironment of the oocyte. This may lead to failure in fertilization. In their study, Munoz et al.^(19)^ reported that the protocols used in stimulation had no effect on embryonic quality but did alter the kinetics of embryonic development. Moreover, we determined no statistically significant difference between the groups in terms of the grades of embryos.

In the present study and two previous studies^(7,8)^, no premature luteinization was observed in any patient even though the GnRH antagonist implementation on the day of hCG was stopped. Moreover, the recent studies showed that the increasing level of progesterone did not decrease pregnancy rates^(20,21)^. For this reason, the results of the present study and those of previous studies suggest that the implementation of GnRH antagonist in stimulation in order to prevent possible negative effects that might develop due to the premature increase of LH deteriorates formation of mature oocyte and the dynamics of embryos’ development. Additionally, the cost of using GnRH antagonists on the hCG day was 5003 USD for the 194 patients in group 2. However, there was no difference in pregnancy rates and there was no premature luteinization in the patients of group 1.

We found no differences in endometrial thickness using transvaginal ultrasonography in patients who did and did not receive antagonist on the day of hCG. Similarly, Chang et al.^(7,8)^ reported that the endometrial thickness, pattern, and implantation rates were not statistically significantly different between patients who did and did not receive GnRH antagonist on the day of hCG. However, high-dose ganirelix was observed to decrease the implantation by causing deteriorated HOXA10 expression in the endometrium^(22)^.

### Study Limitations

The limitation of the study is that the LH and E2 values of the patients were not measured after hCG administration. Thus, we might be able to show that implementation of antagonist on the day of hCG suppresses LH levels and decreases estrogen synthesis. Another limitation is the deficiency of the determination of the implantation rates because of inadequate data.

Although this was a retrospective study, it also incorporated many patients. The implementation of antagonist on the day of hCG within the scope of antagonist protocol might have negative effects on the oocyte maturation and embryonic development. As in previous studies^(7,8)^, no negative consequence of not implementing GnRH antagonist on the day of hCG was observed in the present study.

## Conclusion

In two previous studies^(7,8)^, no difference was determined in pregnancy rates. For this reason, the administration of antagonist on the day of hCG is not acceptable both in terms of costs and excessive injection. Larger sample sized studies are required in order to clearly reveal the effects of GnRH antagonist administration on the day of hCG on embryonic development and oocyte maturation.

## Figures and Tables

**Table 1:**
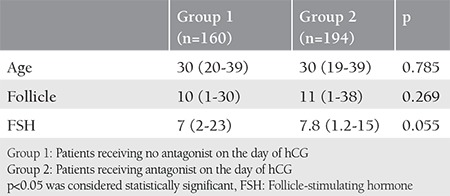
Pre-stimulation evaluation results

**Table 2:**
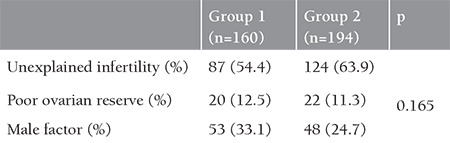
Distribution of patients by the reason of infertility

**Table 3:**
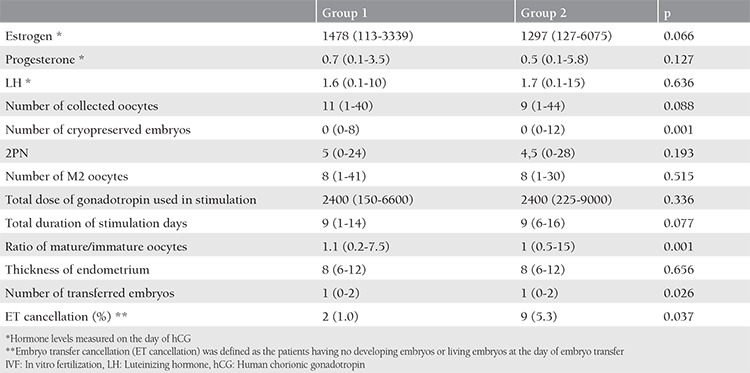
Comparison of controlled ovarian hyperstimulation and IVF-ET findings

**Table 4:**
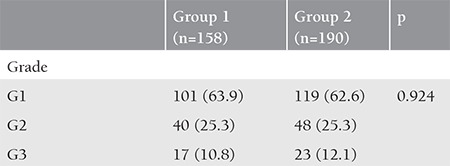
Comparison of embryo grades

**Table 5:**
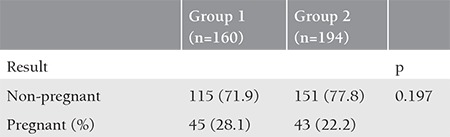
Comparison of the effects on gestational results
